# Intersecting epidemics: immune dysregulation associated with HIV and tuberculosis syndemic contribute to increased risk of hypertensive disease in Sub-Saharan Africa

**DOI:** 10.3389/fcvm.2025.1717609

**Published:** 2025-11-17

**Authors:** Pheletso Letuka, Michael Z. Zulu

**Affiliations:** 1 Division of Cardiology, Cape Heart Institute, University of Cape Town, Cape Town, South Africa; 2 SAMRC Extramural Unit on Intersection of Noncommunicable Diseases and Infectious Diseases, University of Cape Town, Cape Town, South Africa; 3 Department of Virology, School of Pathology, Faculty of Health Sciences, University of Witwatersrand, Johannesburg, South Africa; 4 Institute of Infectious Disease and Molecular Medicine, University of Cape Town, Cape Town, South Africa

**Keywords:** hypertension, immune dysregulation, human immunodeficiency virus (HIV), tuberculosis, chronic infections

## Abstract

Hypertension (HTN) is a chronic medical condition characterized by systolic blood pressure of ≥140 mmHg and diastolic blood pressure >80 mmHg upon repeated measurements. It is one of the most common non-communicable diseases affecting 30% of the global population. Sub-Saharan Africa (SSA) has a high burden of infectious diseases, which contribute to the increased prevalence of hypertension. Furthermore, SSA has the highest number of people living with chronic infectious diseases, such as human immunodeficiency virus (HIV) and tuberculosis (TB). The pathogenesis of these conditions is associated with chronic, low-grade inflammation and immune activation that complicates various homeostatic functions, leading to increased risk of non-communicable diseases among this population. Furthermore, persistent immune activation leads to endothelial dysfunction, arterial stiffness, and altered vascular tone, which contribute to elevated and treatment-refractory blood pressure. However, immunological factors that contribute to the development and pathogenesis of hypertension remain poorly understood. Antiretroviral therapy and anti-TB medications further complicate this landscape by inducing metabolic disturbances and modulating drug metabolism, which affects the efficacy of anti-hypertensive medications. There is a paucity of data and studies reporting on immune dysregulation associated with HTN amongst people living with chronic infections such as HIV and TB. This review aims to highlight this gap in knowledge and the need for more translational research studies to improve health outcomes in hypertensive individuals living with HIV and TB in SSA. Understanding these intertwined immunological and pathophysiological mechanisms is crucial to developing targeted interventions for managing HTN, especially in this vulnerable population.

## 1 Introduction

Hypertension (HTN) is a chronic, cardiovascular condition in which the systolic blood pressure is ≥140 mmHg and/or diastolic blood pressure is >80 mmHg in the arteries upon repeated measurements (1). It is one of the major risk factors for morbidity and mortality, as it contributes to the development of cardiovascular conditions such as heart failure and stroke (2–5). It is one of the most common non-communicable diseases affecting 30% of the global population (6). In 2023, the World Health Organisation (WHO) reported that only 21% of adults with hypertension had their blood pressure controlled (7). The prevalence of hypertension in sub-Saharan Africa (SSA) increased significantly between the years 1999–2019 (8), reaching 34% in men and 48% in women (8, 9). Numerous factors which include increased burden of infectious disease, changes in diet, lifestyle and poor healthcare are contributing factors to this (8, 10–13). Key factors associated with the high prevalence of hypertension and its disparities across sub-Saharan Africa include dietary patterns (8, 14–16), abnormal body weight (both increased adiposity and underweight) (8, 17–19), population ageing (8, 18, 20–22), socioeconomic status (education and income), and psychosocial stressors (8, 14, 17, 20, 21). Infectious diseases such as coronavirus disease 2019 (COVID-19) (23, 24), human immunodeficiency virus (HIV) (25, 26) and tuberculosis (TB) (27) have also been implicated in the development of hypertension. Resistant hypertension (RH) is defined as BP that remain elevated despite the concurrent administration of three or more antihypertensive agents of different classes, including a diuretic (28). The exact aetiology of resistant hypertension is unknown; however, it is postulated to be associated with multiple factors such inappropriate treatment and limited access to healthcare, high-salt diet, volume overload and hyperactivation of the sympathetic nervous system (29, 30).

The highest number of people living with chronic infections such as HIV and TB is in SSA (31, 32). People living with HIV (PLWHIV) and TB are at an increased risk of developing hypertension (33, 34) (Table 1). The high burden of chronic infections in SSA poses a significant public health challenge as weakens the already under-resourced healthcare system and increases the risk of non-communicable disease in the population (34–37). Underlying conditions such as HTN pose a significant public health challenge around the world (33, 34, 38). Furthermore, the mechanisms affecting pathogenesis of infectious disease in people living with chronic infections remains poorly understood. Therefore, there is a paucity of data and research studies on immune mechanisms associated with development of hypertension amongst people living with chronic infections such as HIV and TB. This review aims to highlight this gap in knowledge and the need for more translational research studies to improve health outcomes in hypertensive individuals living with HIV and TB in SSA. Understanding these intertwined immunological and pathophysiological mechanisms is crucial to developing targeted interventions for managing HTN, especially among this vulnerable population.

**Table 1:** Prevalence of HTN amongst different population groups living with chronic infections in SSA.

Group	Prevalence	Prevalence (Stratified by age)	ART-specific effects	Adjusted effect sizes	References
People living with HIV (PLWHIV)	Pooled prevalence (SSA): 21.9% (95% CI 19.9%–23.9%). Pooled mean BP ≈ 120/77 mmHg.	>50% in PLWH ≥50y; ∼31%–41% (<35–44y) rising to 54%–58% (≥45y) in South African cohorts.	ART exposure aPR ≈ 1.23 (Chen et al.); INSTI regimens linked with higher HTN risk (aIRR 1.7–1.9 vs NNRTIs).	Male sex aPR 1.33; ART aPR 1.23; CD4 ≥ 200 aPR 1.45 (Chen et al.); INSTI vs NNRTI aIRR 1.76–1.92 (Byonanebye et al.).	(108)
Active TB (patients with TB disease)	Very heterogeneous; reported 0.7%–38% across older studies; limited high-quality evidence.	Older TB patients more likely to have HTN; data sparse and variable across studies.	N/A (ART only relevant in co-infection).	Systematic review: no consistent adjusted association (Seegert et al., 2017).	(35)
Latent TB infection (LTBI/TBI)	NHANES (US): overall HTN prevalence ≈ 48.9%; higher crude prevalence in LTBI (58.5% vs 48.3%) but attenuated after adjustment.	Crude HTN prevalence higher in LTBI across all age groups; adjusted analyses show similar rates after controlling for age.	N/A (LTBI typically not treated with ART).	Adjusted PR ≈ 1.0 (95% CI 0.9–1.1) after controlling for age, sex, BMI, diabetes, smoking (Salindri et al.).	(27)
HIV–TB co-infected (prospective cohorts)	South Africa (SAPIT/TRuTH cohort): incidence ≈ 1.9 per 100 person-years; men ≈ 5.9, women >40y ≈ 5.0 per 100 PY.	Incidence sharply higher with age and sex: men 5.9/100 PY; women >40y 5.0/100 PY.	ART initiation post-TB associated with weight gain and increased HTN risk, especially with INSTI-based regimens.	Adjusted HRs: men aHR 12.04; women >40y aHR 8.19; men <40y aHR 2.79 (Dawood et al., 2024).	(138)

## 2 Inflammation and hypertension

**Figure 1:**
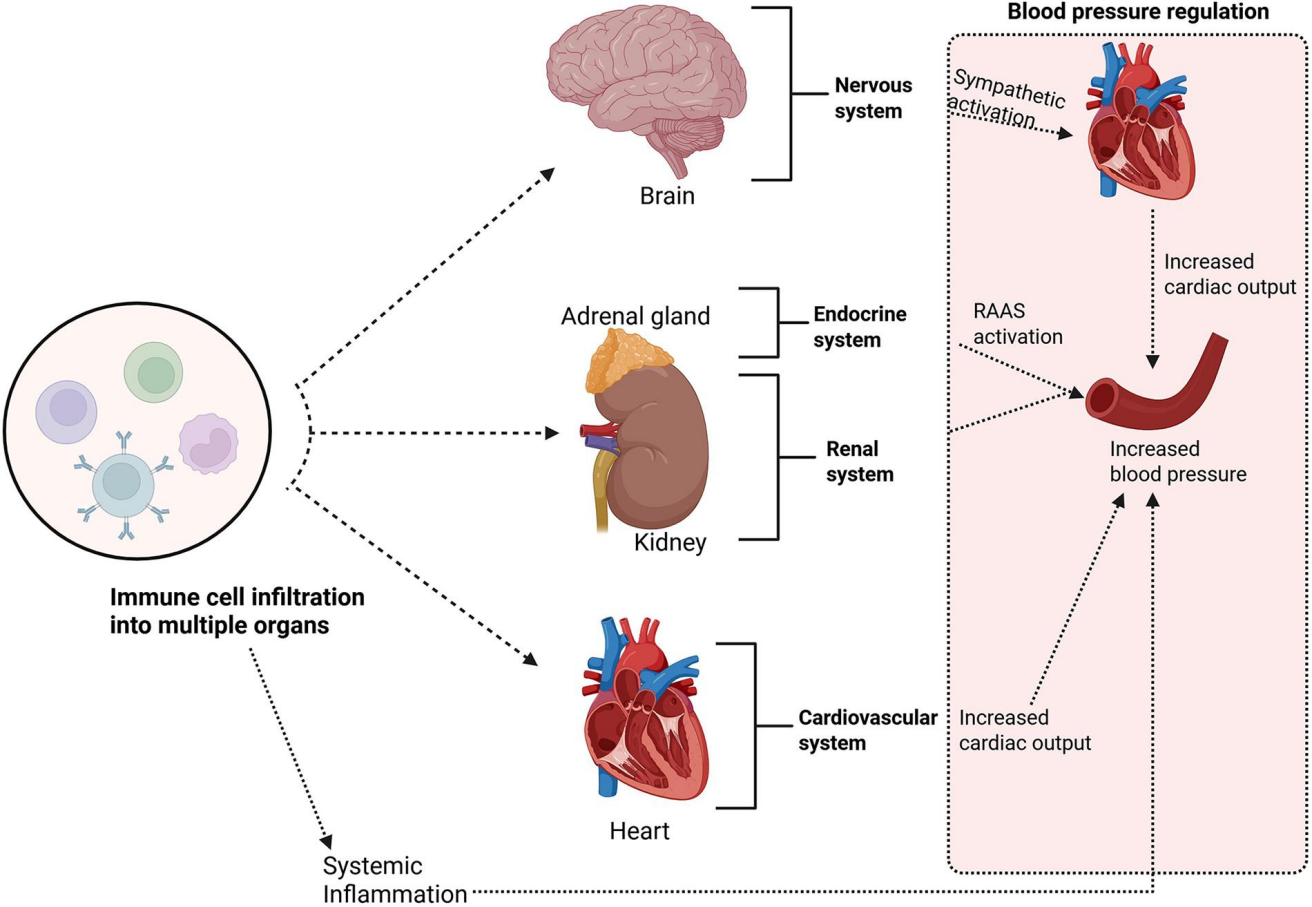
Immune cells infiltrate organs critical to blood pressure regulation, such as the blood vessels, kidneys, heart, and brain; leading to chronic inflammation. This chronic inflammatory state disrupts normal organ function and contributes to the onset and progression of hypertension. *Created using Bio-render*.

Inflammation plays a crucial role in the pathogenesis of hypertension (39–42). It has been shown that T lymphocytes are required for the full development of angiotensin II– and DOCA-salt–induced hypertension and vascular dysfunction in mice; T cells (*via* AT1 receptor and NADPH oxidase–dependent ROS and cytokine production) infiltrate perivascular tissue and drive vascular oxidative stress, endothelial dysfunction and blood-pressure elevation (43). Immune activation in hypertension is kick-started by well-known pro-hypertensive stimuli such as, increased sympathetic outflow (44, 45), acute and chronic stress (46), excessive salt intake (47, 48), gut microbial dysbiosis (49, 50), local oxidative-stress (51) and vascular dysfunction (52). A key concept is that immune cells infiltrate organs critical to blood pressure regulation, such as the blood vessels (53), kidneys, heart (54), and brain, leading to chronic inflammation (43, 52, 55, 56). This chronic inflammatory state disrupts normal organ function and contributes to the onset and progression of hypertension (52). The inflammatory and immune mechanisms driving hypertension are regulated through a complex interaction between innate and adaptive immune cells (Figure 1) (52). The early development of hypertension involves activation of innate immune cells, such as macrophages, dendritic cells, and natural killer (NK) cells, which initiate inflammatory responses by releasing cytokines and chemokines (51, 52, 57). These signalling molecules facilitate the recruitment of additional immune cells, including T and B lymphocytes, to the site of inflammation (52). Once activated, T cells can differentiate into effector subsets; such as T helper 1 (Th1) and Th17 cells; which release cytokines that amplify the inflammatory response and contribute to vascular dysfunction (52, 58, 59).

## 3 Immunity and hypertension

Hypertension has long been associated with activation of immune pathways (2), here we discuss the cells of the innate and adaptive immune system and their role in hypertension development. Dendritic cells (DCs) are specialised antigen-presenting cells (APCs) derived from hematopoietic progenitors in the bone marrow and are widely distributed across various tissues (60, 61). They possess a unique capacity to initiate T cell responses by capturing, processing, and presenting antigens to naïve T cells. Therefore, DCs serve as key orchestrators of the adaptive immune system (60, 61). DCs from hypertensive mice exhibit elevated surface expression of the B7 ligands CD80 and CD86, indicative of DC maturation and activation (51, 62). Inhibition of these costimulatory molecules has been shown to prevent the development of hypertension and the activation of T cells in models of both angiotensin II- and DOCA-salt induced hypertension (51, 62). In mice, oxidative stress in hypertension generates reactive γ-ketoaldehydes known as isoketals, which accumulate in and activate DCs to drive T-cell responses that contribute to elevated blood pressure (51). Plasma levels of F2-isoprostanes, oxidative stress markers produced alongside isoketals, were elevated in individuals receiving treatment for hypertension and significantly higher in those with resistant hypertension (51). Additionally, isoketal-modified proteins were notably increased in circulating monocytes and dendritic cells from hypertensive patients (51). These findings suggest a positive feed-forward loop between hypertension and DCs.

Over the years, monocytes and macrophages have been associated with hypertension development (55). This is evidenced by notable increases in their numbers and alterations in their phenotype observed within the vasculature, kidneys, heart, and brain across various models of hypertension, in comparison to normotensive controls (55, 63–68). In humans, macrophage subsets are grouped by the expression of CD14 and CD16. This breaks the macrophage populations into 3 subsets: CD14++/CD16−−(“classical monocytes or macrophages”), CD14+/CD16+ or CD14++/CD16+ (“intermediate monocytes or macrophages”), and CD14dim or +/CD16++ (“nonclassical monocytes or macrophages”) (69, 70). CD14+ and CD16+ macrophages are taken to be correlates of the M1 and M2 phenotypes (69). Directly implicating CD14+ human macrophages in the development of clinical hypertension is their robust expression of angiotensin-converting enzyme (69, 71), demonstrating their potential involvement in hypertension through participation in the renin-angiotensin-aldosterone system (RAAS), which may lead to feedforward activation of monocytes and “switching” between the M1 and M2 phenotypes (69). There are less studies on humans but the data that are available support a role for inflammation and macrophage polarization in essential hypertension, as well as cardiovascular disease (69). One study showed that the severity of hypertension was linked to the presence of CD68+ M1 inflammatory macrophages in the kidney, independent of race (69, 72). This indicates a possible role of macrophages in the development of hypertension.

Various subsets of T lymphocytes influence blood pressure regulation by modulating the local cytokine environment within cardiovascular regulatory organs (73). Hematopoietic stem cells in the bone marrow differentiate into naïve T cells which mature in the thymus before entering systemic circulation and migrating to distant tissues (73, 74). Single CD4+ T cells are classified as T helper cells (Th cells), CD8+ T cells are referred to as cytotoxic T cells, and CD1d+ T cells are known as natural killer T cells (73). Once an antigen is presented via the major histocompatibility complex (MHC) and binds to the T cell receptor (TCR) on the naïve CD4+ T cell, the T cell differentiates into distinct T helper (Th) lineages e.g., Th1, Th2, Th17, or T regulatory (Treg) in response to the local concentrations of specific cytokines. These T cell subsets provide helper functions by secreting specific cytokines that target other immune cells and modulate both vascular reactivity and renal sodium handling (73). Immune activation in hypertension is characterised by activation of T-cells via DC activation (75). T-cells then migrate to the vascular tree and the kidney causing inflammation and hypertension therefore, factors effecting T-cell activation and function are important mediators of essential hypertension (75). In an attempt to characterise T cells in newly diagnosed, treatment-naïve hypertensive individuals by assessing circulating levels of C-X-C chemokine receptor type 3 (CXCR3) chemokines, findings revealed that hypertensive patients had a higher proportion of immunosenescent, proinflammatory, and cytotoxic CD8^+^ T cells compared to healthy controls (76). Similarly in a different study, the frequency of both CD4+ and CD8+ CD45RO+ (memory) circulating T cells was higher in the hypertensive patients than in the normotensive controls (54). Hypertensive patients exhibited a higher frequency of CD4+ 1l-17A+ compared to normotensive controls. Interferon gamma (IFN-*γ*) and tumour necrosis factor-alpha (TNF-α) were also increased in CD4+ T cells, and CD8+ T cells of hypertensive patients (54). These results demonstrate the role of T-cell driven inflammation in mediating hypertension.

## 4 Immunity and HIV/TB

### 4.1 Immune dysregulation and the human immunodeficiency virus

Human immunodeficiency virus (HIV)) is an infection that targets and damages the immune system, specifically the CD4+ white blood cells that play a crucial role in immune defence (77). For successful entry, the virus must engage both the CD4 receptor and one of two co-receptors on the host cell surface—the C-C chemokine receptor type 5 (CCR5) or the C-X-C chemokine receptor type 4 (CXCR4) (78–80). Beyond CD4^+^ cells, HIV infects macrophages and dendritic cells, disrupting antigen presentation and cytokine networks (78). By destroying these cells, HIV progressively weakens the body's ability to fight off opportunistic infections such as tuberculosis (77). The combination of persistent viral replication, mucosal barrier breakdown, and innate immune sensing (*via* Toll-like receptors and inflammasomes) sustains a state of chronic inflammation and immune activation, which paradoxically accelerates immune exhaustion and CD4^+^ decline despite antiviral responses (81).

Despite effective antiretroviral therapy (ART), immune dysregulation continues as a result of persistent latent viral reservoirs and sustained low-level immune activation (82). T-cell exhaustion, characterized by increased expression of inhibitory receptors such as PD-1 (83, 84), TIM-3, and LAG-3 (84), diminishes HIV-specific immune responses (85), while dysregulated cytokine production drives inflammation and immune activation (86, 87). Chronic inflammation accelerates immunosenescence and promotes the development of non-communicable diseases, including cardiovascular disease and certain cancers. Therefore, immune dysregulation in HIV extends beyond CD4^+^ T-cell depletion, representing a complex disorder characterized by sustained immune activation, impaired regulatory mechanisms, and incomplete immune recovery, even in the context of effective ART (88, 89).

### 4.2 Immune activation in Tuberculosis

Tuberculosis (TB) is a chronic infectious disease caused by the bacterium *Mycobacterium tuberculosis* (M.tb) (90). It primarily affects the lungs (pulmonary TB) but can also involve other organs (extrapulmonary TB) (90). TB is transmitted through airborne particles when an infected person coughs, sneezes, or speaks. Once inhaled, the bacteria can remain dormant (latent TB) or progress to active disease, especially in individuals with weakened immune systems (90). The innate immune system serves as the initial defense against M.tb infection (91). This largely depends on initial interactions with host innate immune cells, such as macrophages, dendritic cells, neutrophils, and natural killer cells (92). Multiple innate-like leukocytes also contribute to the host defense against M.tb, including non-conventional T-cell subsets such as mucosal-associated invariant T (MAIT) cells, CD1-restricted T lymphocytes, and natural killer T (NKT) cells (92). The initiation of innate immune responses to M.tb infection begins with pathogen recognition. During phagocytosis, conserved pathogen-associated molecular patterns (PAMPs) on the M.tb surface are detected by pattern recognition receptors (PRRs) expressed on host immune cells (92). In addition to phagocytosis, autophagy, apoptosis and the inflammasome activation, innate immune cells also trigger inflammatory cytokine and chemokine production to eliminate invading pathogens (93). M.tb manipulates host immune and metabolic pathways to evade clearance and establish persistent infection. Early studies in murine models, subsequently corroborated in humans (94), identified IFN-γ, TNF, and IL-1β as key cytokines required for effective immune control of M.tb (95–98). Signalling through the IL-1 receptor is modulated by the IL-1 receptor antagonist (IL-1ra), which competitively inhibits IL-1 binding (99). Elevated circulating concentrations of IL-1ra have been reported in individuals with active tuberculosis and proposed as a potential biomarker for disease activity and therapeutic response (100). Nonetheless, the molecular mechanisms underlying increased IL-1ra expression during active TB remain poorly understood.

## 5 Discussion

### 5.1 Intersection between infectious disease and non-communicable diseases

**Figure 2:**
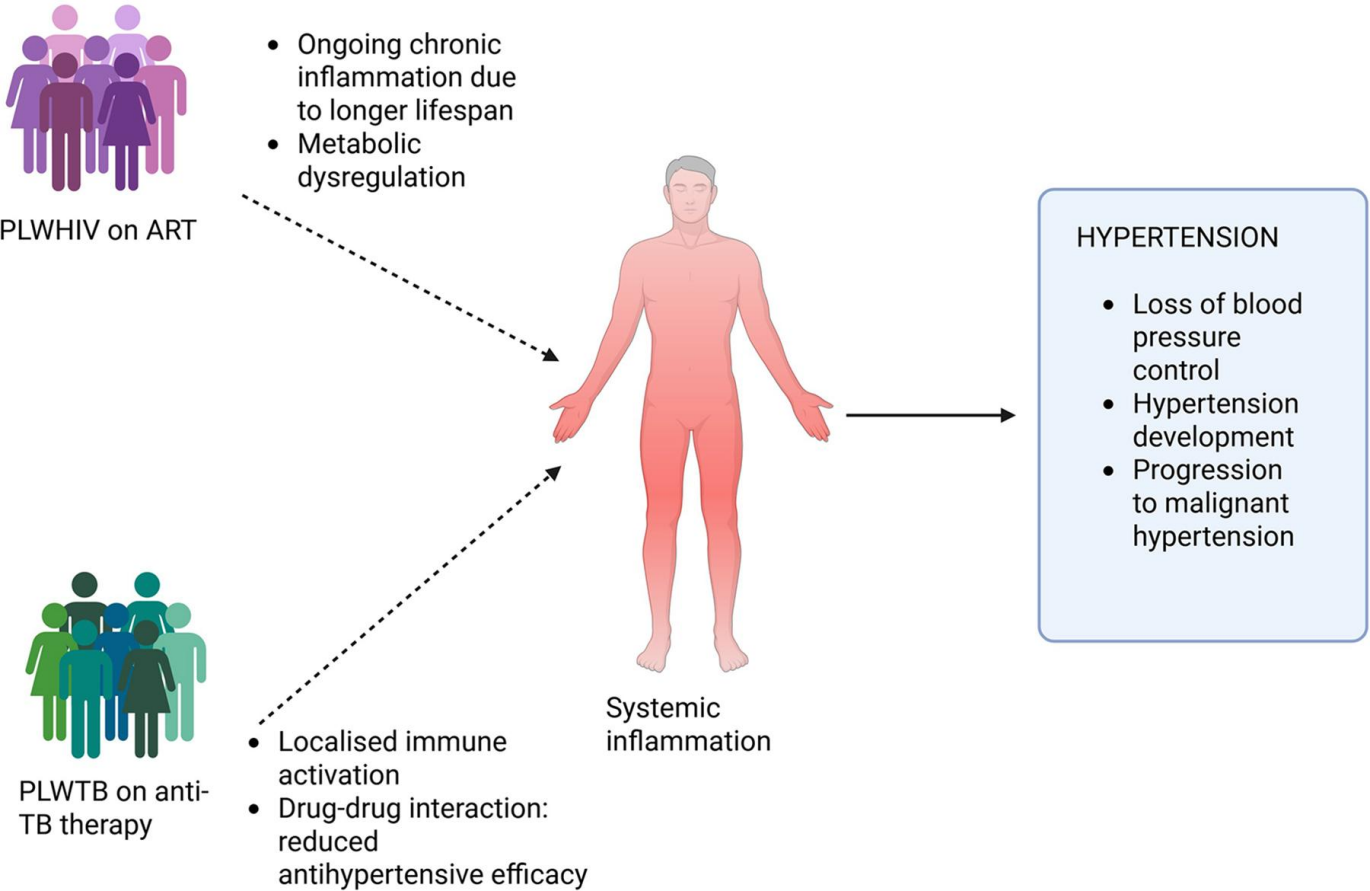
Chronic immune activation, inflammation, endothelial dysfunction, and metabolic derangements are induced by HIV infection, TB, and their respective treatments. ART is associated with metabolic complications which increase the risk of hypertension. Similarly, anti-tuberculosis medications, such as rifampicin, can interfere with the pharmacokinetics of commonly used antihypertensive drugs leading to subtherapeutic drug levels and poor blood pressure control. *Created using Bio-render*.

An estimated 40.8 million people were living with HIV globally at the end of 2024, with only 77% receiving antiretroviral therapy (ART) (36). The SSA region carries the greatest number of PLWHIV, in 2019, approximately 26 million individuals in SSA were living with HIV (32). Moreover, SSA accounted for 670,000 of the 1.5 million new HIV infections and 280,000 of the 650,000 AIDS-related deaths reported globally in 2021 (101). Hypertension is an increasingly common concern among adults living with HIV, particularly those receiving ART (102). While ART has significantly improved survival outcomes for PLWHIV, it has also been associated with an increasing burden of cardiovascular disease (103–106) (Figure 2). The pooled hypertension prevalence in PLWHIV in SSA was 21.9% alongside mean systolic blood pressure/diastolic blood pressure levels of 120/77 mmHg, withs significantly higher hypertension prevalence among males, ART users, and individuals with CD4 counts ≥200 cells/mm^3^ (107). These results underscore the need for cardiovascular risk integration into HIV care, especially as ART access and life expectancy continue to rise. The underlying mechanisms driving hypertension and cardiovascular disease in PLWHIV remain incompletely understood (103). In one study, the prevalence of hypertension among PLWHIV in SSA varied widely, ranging from 2.0%–50.2%, with most cases observed in individuals receiving ART (103). A retrospective Zambian cohort of PLWHIV initiating ART found that males developed hypertension earlier (2 years) compared to females (6 years) after ART initiation. In multivariable analysis, higher baseline SBP/MAP and use of certain ART (protease inhibitors) predicted incident hypertension in males but not in females (108).

Most studies report the prevalence of hypertension in PLWHIV, while data on the detailed mechanisms of how HIV and TB dysregulate the immune activation in hypertension remain scarce. Nonetheless, a mechanistic approach to how HIV dysregulates immune activation in the setting of hypertension can be summarised in 4 steps. (1) *Chronic immune activation and pro-inflammatory cytokines:* In addition to traditional risk factors and the impact of ART on blood pressure, inflammatory markers such as IL-17A, IFN-γ, and CD4^+^ T cells have been linked to hypertension in PLWHIV undergoing ART (103). The same authors propose that HIV viral proteins and ART interact with the immune system to synergistically contribute to kidney injury, vascular dysfunction, and alterations in sympathetic nervous activity, ultimately promoting the development of hypertension (103). Similarly, in another study assessment of inflammatory biomarkers in plasma demonstrated an association between HIV-induced inflammation and increased blood pressure levels (109). In an attempt to investigate how T-cell activation/exhaustion and monocyte subsets correlate with arterial stiffness, the authors noted that individuals with HIV who displayed elevated levels of PD-1+ exhausted CD4+ and CD8+ T cells showed evidence of stiffer arteries early in ART treatment (110). (2) *Microbial translocation, monocyte activation and endothelial injury:* HIV damages gut mucosa early on, allowing lipopolysaccharides (LPS) into the circulation (111). LPS/sCD14-mediated monocyte activation sustains systemic inflammation and directly impairs endothelial function (112), linking microbial translocation to vascular dysfunction and hypertension risk. (3) *Viral proteins and immune cell-vascular cross talk:* HIV proteins and viral-protein-expressing immune cells can act on endothelial and smooth muscle cells to increase oxidative stress, inflammasome activation, and local cytokine production, promoting vasoconstriction and remodelling (103). Recent work using the Tg26 mouse model of HIV shows CD4+ T cells expressing viral proteins can drive hypertension through IL-1α and NOX1 pathways (113)*.* (4) *Monocyte/macrophage and T-cell phenotypes that favour hypertension:* HIV shifts innate and adaptive cell populations toward activated, inflammatory phenotypes (pro-inflammatory monocytes, senescent/activated CD8+ T cells, dysfunctional Tregs) (103). These cells infiltrate kidneys and vessels, producing cytokines and ROS that raise systemic vascular resistance and alter renal sodium handling (103, 113).

There is limited evidence regarding whether HIV infection contributes to an increased risk of resistant hypertension (RH) (114). A case–control study in Malawi (114) has been designed to evaluate these associations, though findings are not yet available. Current research on HIV–associated hypertension predominantly centres on cardiovascular disease mechanisms in PLWHIV; therefore, the precise contributors to hypertension remain poorly characterized. Comprehensive studies are required to assess whether the same biological pathways underlying HIV-related CVD are responsible for increased blood pressure. In addition, large multinational longitudinal cohorts are needed to define the mechanisms and predictors of hypertension in PLWHIV relative to people living without HIV (PWoH). The mechanisms linking immune activation or inflammation to hypertension and RH in the context of HIV remain poorly understood and require further investigation.

Although the global burden of TB is declining, it remains a significant public health challenge in many low- and middle-income countries (LMICs) (34). In 2021, 10.6 million individuals developed TB globally and an estimated 1.6 million people died from the disease (115, 116). LMICs accounted for 80% of all TB cases and deaths, with the WHO African region contributing 23% of new infections (115, 116). TB ranks as the second deadliest infectious disease and the 13th leading cause of death worldwide (115, 116). TB has been implicated in the pathogenesis of hypertension through diverse immunological and pathophysiological mechanisms (34). While there is no specific data on immune mechanisms in TB and hypertension in SSA, or globally, many believe that activation of immune responses in TB may impair endothelial function (Figure 2), thereby increasing the risk of cardiovascular disease and potentially contributing to the development of hypertension (34, 117, 118). TB may cause pulmonary hypertension by damaging the pulmonary vessels (34, 119), and may cause systemic hypertension via TB infection in the kidney, thereby causing damage to the renal tissue, decreased renal function, and impaired blood pressure regulation (34, 120, 121).

There are plausible, partly well-worked mechanisms by which active or latent M.tb infection can dysregulate immunity in ways that increase blood-pressure and cardiovascular risk. The evidence is a mix of epidemiology, clinical case reports, immunology, and animal/biomarker studies; causal chains are biologically plausible but not yet proven in randomized trials. M.tb elicits strong cellular immunity (IFN-γ, TNF-α, IL-1 family, IL-6) (118). Persistent or recurrent antigen exposure (active disease, poorly controlled latent infection, or post-treatment immune remodelling) raises circulating pro-inflammatory cytokines (122) that are known drivers of vascular inflammation, endothelial dysfunction, arterial stiffness and BP elevation.

Inflammation and immune activation in TB further complicate the management of hypertension. TB, as a chronic inflammatory condition, may trigger a complex sequence of immune responses that contribute to the development of atherosclerotic plaque formation (117). This process involves infection-induced antibodies cross-reacting with self-antigens, including heat-shock proteins (HSPs), which are a family of stress-responsive proteins expressed by cells under various physiological stress conditions (123). Human HSP60 exhibits approximately 40%–50% identical resemblance with heat-shock proteins found in *Mycobacterium* species (118). A similar mechanism may underlie the development of hypertension in individuals with TB (118). It has been shown that overexpression of HSPs can provoke autoimmune responses, resulting in the infiltration of macrophages and T-lymphocytes into renal tissue; an effect linked to hypertension in experimental models (118). Additionally, patients with essential hypertension have been found to exhibit elevated levels of anti-HSP70 and anti-HSP65 antibodies (118).

Immune activation in TB may exacerbate the inflammatory milieu that underlies hypertension by contributing to vascular dysfunction and elevated blood pressure (Figure 2). Epidemiological data further suggest a heightened risk of hypertension among individuals with latent TB, linking infectious disease status with chronic cardiovascular risk. At present, there is no data to suggest TB contributes to the development of RH, however it is not rare to come across cases where TB presents itself as malignant or uncontrolled hypertension (124).

### 5.2 Impact of ART and anti-TB medication on pathogenesis of hypertension

HIV treatment relies on combination ART to improve therapeutic outcomes (77). The widespread success of ART has been accompanied by a rising prevalence of NCDs among PLWHIV (125). As individuals with HIV experience longer lifespans due to effective ART, chronic comorbidities such as hypertension, have become prominent contributors to morbidity and mortality in this population (126–128). PLWHIV who are receiving combination ART have a higher risk of developing hypertension compared to those without HIV infection (129, 130). Prolonged use of highly active antiretroviral therapy (HAART) (beyond two years) is associated with a significantly increased risk of systolic hypertension, even after controlling for age, body mass index, race, and smoking (131). Other cardiovascular risk factors mediated by ART include hypertriglyceridemia, hypercholesterolemia, and atherosclerosis (132), well known risk factors for hypertension (133, 134).

The WHO endorses the use of fixed-dose combination regimens for anti-tuberculosis therapy, comprising isoniazid, rifampicin, pyrazinamide, and ethambutol as the standard first-line treatment (135). Rifampicin may reduce the efficacy of various antihypertensive medications by inducing cytochrome P450 enzymes, thereby accelerating their metabolism (136). The effects of rifampicin on blood pressure control and antihypertensive drug levels in 24 hypertensive patients with end-stage chronic kidney disease undergoing maintenance haemodialysis was studied. All participants had stable blood pressure (≤140/90 mmHg) on consistent antihypertensive regimens before starting rifampicin-based treatment for TB (137). However, after rifampicin initiation, there was a significant decrease in plasma concentrations of commonly used anti hypertensives. This decrease in levels correlated well with worsening of hypertension (137).

## 6 Conclusion

The growing intersection between infectious diseases and non-communicable conditions represents one of the most pressing challenges for health systems in SSA. Among PLWHIV and TB, the emergence and persistence of hypertension and resistant hypertension have significant implications for long-term morbidity and mortality. This complex clinical overlap is driven by a convergence of factors, including chronic immune activation, inflammation, endothelial dysfunction, and metabolic derangements induced by HIV infection, TB, and their respective treatments. ART, while lifesaving, is associated with metabolic complications which increase the risk of hypertension. Similarly, anti-tuberculosis medications, most notably rifampicin, can interfere with the pharmacokinetics of commonly used antihypertensive drugs, leading to subtherapeutic drug levels and poor blood pressure control. Moreover, HIV and TB themselves exert profound immunological effects that may contribute to vascular inflammation and endothelial injury, further complicating the pathophysiology of hypertension in co-infected individuals.

In SSA, these challenges are compounded by systemic issues such as limited access to diagnostics, poor integration of care for infectious and chronic diseases, and fragmented health service delivery. The traditional disease-specific focus of public health programs has left a critical gap in managing comorbid non-communicable diseases in populations with high burdens of HIV and TB. As a result, hypertension often remains undiagnosed or poorly controlled, increasing the risk of cardiovascular events, renal disease, and premature death. To mitigate this growing burden, there is an urgent need for integrated models of care that combine infectious disease management with routine screening and treatment of non-communicable diseases, including hypertension. Future research should prioritize understanding the immunopathogenic mechanisms linking HIV, TB, and hypertension, and explore context-appropriate strategies to overcome drug–drug interactions and improve therapeutic outcomes. Strengthening health systems to provide holistic, continuous, and coordinated care will be essential in addressing this emerging syndemic and improving long-term outcomes for affected populations across sub-Saharan Africa.
